# OneZoom: A Fractal Explorer for the Tree of Life

**DOI:** 10.1371/journal.pbio.1001406

**Published:** 2012-10-16

**Authors:** J. Rosindell, L. J. Harmon

**Affiliations:** 1 Division of Biology, Imperial College London, Silwood Park Campus, Ascot, Berkshire, United Kingdom; 2 Department of Biological Sciences and Institute for Bioinformatics and Evolutionary Studies (IBEST), University of Idaho, Moscow, Idaho, United States of America

## Abstract

An intuitively simple zooming interface offers a visually appealing way to explore very large phylogenetic trees with metadata.

## We Can't See the Trees for the Data

Our knowledge of the tree of life—a phylogenetic tree summarizing the evolutionary relationships among all life on Earth—is expanding rapidly. “Mega-trees” with millions of tips (species) are expected to appear imminently (for example, see http://www.opentree.wikispaces.com). Unfortunately, there has so far been no practical and intuitive way to explore even the much smaller trees with thousands of tips that are now being routinely produced. Without a way to view megatrees, these wondrous objects, representing the culmination of decades of scientific effort, cannot be fully appreciated. The field really needs a solution to this problem to enable scientists to communicate important evolutionary concepts and data effectively, both to each other and to the general public.

Just like Google Earth changed the way people look at geography, a sophisticated tree of life browser could really change the way we look at the life around us . . . Our advances in understanding evolution are moving really fast now, but the tools for looking at these big trees are lagging behind. (Westneat in [1], February 2009)

Displaying large trees is a hard problem that has so far resisted solution. We are still waiting for the equivalent of a Google Maps. (Page in [2], June 2012)

In this manuscript, we introduce a new approach that solves the problem. Trees with millions of tips, richly embellished with additional data, can now be easily explored within the web browser of any modern hardware with a zooming user interface similar to that used in Google Maps.

## Escaping the Paper Paradigm

Much of the difficulty with phylogenetic tree visualization (and with data visualization more generally) is that we all too often constrain ourselves to the “paper paradigm”—the practice of displaying data in ways that are optimized for printing on paper. Many applications fail to take full advantage of the freedom that a computer display gives us over printed sheets; we read and write documents and browse web pages that are constrained to be optimal for printing, but fail to realize that such documents are unlikely to be optimal for visualization on a digital device. Of course, the paper paradigm of visualization seems most natural to us, but is this only because we are too familiar with the paper format? Society is undergoing a rapid transition in terms of its use of computing devices. Computers have enabled us to generate and store large amounts of data that would not have been possible using paper. We now need to take the next step with a transition to data visualization that is optimized for interactive displays rather than printed paper.

Recent methods of phylogenetic tree visualization attempt to buy extra space in the paper paradigm—for example, by using walls consisting of multiple displays (see Figure 7a in [3]). This approach is costly and does not give tree visualization capabilities to the masses, which is what is really needed. Furthermore, expensive display technology does not really solve the problem—according to our estimates, even the most advanced technology, such as NASA's “Hyperwall2” of 128 LCD displays [4], would not be large enough to clearly display 5,000 tip trees using conventional techniques. Other currently available methods make exploration of phylogenetic trees interactive, enabling the user to expand or magnify parts of the tree [5] that may be too small to see in detail at the scale of the screen. Hyperbolic tree browsers [6],[7] are a good example of this and they can display large trees, but users do not find them intuitive [3] and we don't see the inclusion of rich metadata as being realistically achievable. An optimal tree viewer should be able to 1) handle large megatrees; 2) be explored in an intuitive way; 3) incorporate significant amounts of metadata; and 4) be visually appealing and immersive, especially if public users are expected. We have yet to find an existing viewer that we feel convincingly meets these requirements.

## Zooming in on a Potential Solution

**Figure 1:**
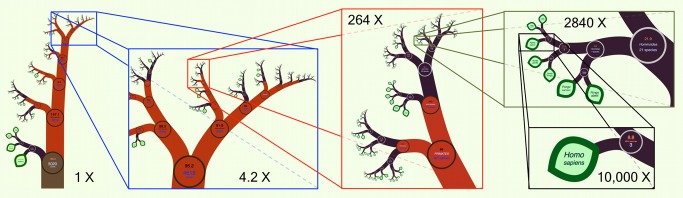
An example IFIG of mammals compiled from the data in [**8**].

**Figure 2:**
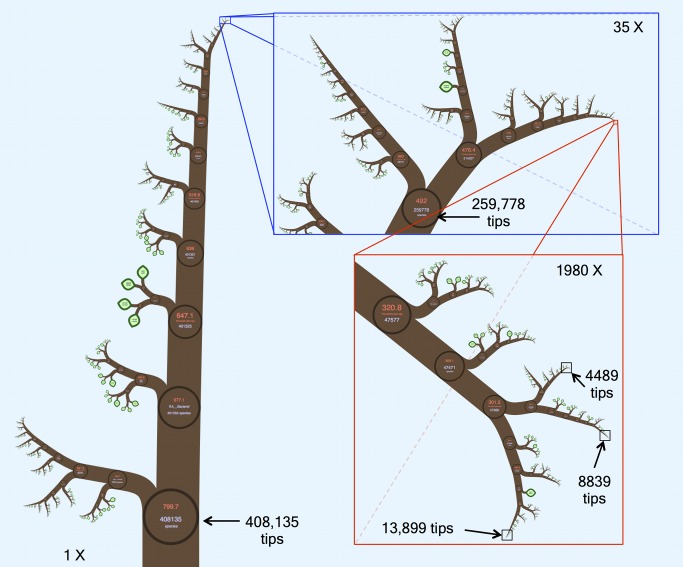
An example IFIG of a 408,135-tip phylogeny of small-subunit RNAs using data from SILVA [**9**].

We introduce a new phylogenetic tree viewer that allows interactive display of large trees. The key concept of our solution is that all the data is on *one* page so that all the user has to do is *zoom* to reveal it—hence the name OneZoom (http://www.onezoom.org). Our interface is analogous to Google Earth, where one can smoothly zoom into any local landmark from a start page showing the whole globe, recognizing familiar landmarks at different scales along the way (e.g., continents, countries, regions, and towns). Equivalently, OneZoom can zoom smoothly to one tip of the tree of life—say, human beings—passing the familiar clades of animals, vertebrates, mammals, and primates at different scales along the way (see Figure 1, which used data from [8]). Trees with millions of tips may require a page of paper larger than the observable universe to be printed: they break the paper paradigm, but on screen, users can still easily zoom in through the tree to any point of interest (see Figure 2, which used data from [9]).

In order to place the phylogenetic data on a scalable two-dimensional map, OneZoom uses concepts inspired by fractal geometry. Fractals are objects that look similar at different scales and have a dimension that is not a whole number; they often appear in the natural world [10],[11]. For example, an effective lung requires a large surface area to be packed into a small a volume—its surface is therefore so convoluted and labyrinthine that at some scales it can be regarded mathematically as having a dimension greater than two (a surface) but less than three (a solid object). Fractal geometry can be used to produce stunning and apparently complex images—including many that resemble trees and other plants—based on remarkably simple sets of underlying growth rules that are applied repeatedly (“L-systems” [12]). Our algorithms (Text S1) build on this to produce a zoomable object, inspired by fractals, but embellished with useful data (such as images, graphs, and text) at every scale—an interactive fractal-inspired graph (IFIG). We give the branches of the tree a width so that they appear as filled shapes rather than narrow lines and thus can contain rich metadata within them that is easily accessible through zooming. Ultimately, these methods should make it possible to usably view the complete tree of life together with all its metadata on any modern computing device (Box 1 and Software S1, S2).

**Box 1. Features of OneZoom**

***Fractal structures***

**Figure 3:**
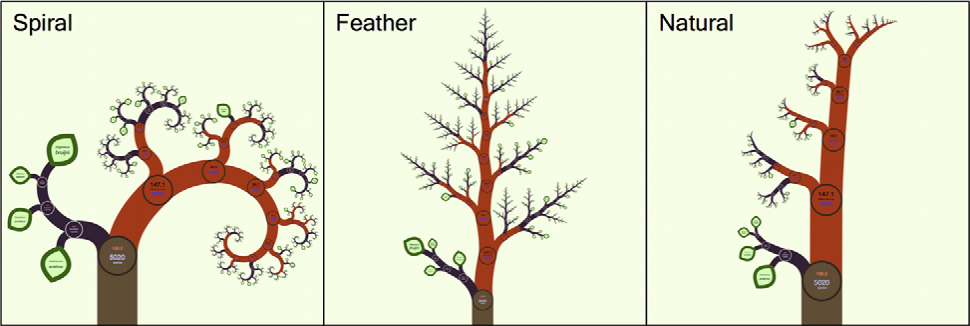
The different fractal forms available in OneZoom.

By varying the angles between the branches and the successive ratios of branch lengths and widths, many different fractal structures are possible for an IFIG of a phylogenetic tree (Figure 3). By allowing these to be defined uniquely at each split according to the tree's properties, the balance can be seen at a glance for every part of the tree.

***Time scales and dated trees***

Many phylogenetic trees also include branch lengths, which provide information about the time scale of evolution. Our software plots the phylogeny in a shape that can reflect the topology and balance, whilst the timescales are expressed in ways that are independent of shape including color, text, and animation. In future work it should be possible to express timings with additional new fractal forms.

***Polytomies***

Phylogenetic trees frequently include polytomies, which are nodes with more than two descendent branches. These generally represent phylogenetic uncertainty in the relationships among clades. Our software can highlight the nodes that make up polytomies in one of a number of ways, or, alternatively, it can not draw branches of length 0 so that polytomies appear as physical breaks. In the future, it should be possible to display polytomies as a number of branches joining at a single point.

***Search functionality***

In a tree of the proportions OneZoom can visualize, it is necessary to have an efficient search function so that users can find leaves or clades easily. OneZoom's search function ranks clades according to the total number of search hits and the proportion of tips in a clade that represent a hit. Search results can be highlighted on the tree or shown instantly. OneZoom also allows users to fly through the tree to search hits with an animation feature.

***Metadata***

**Figure 4:**
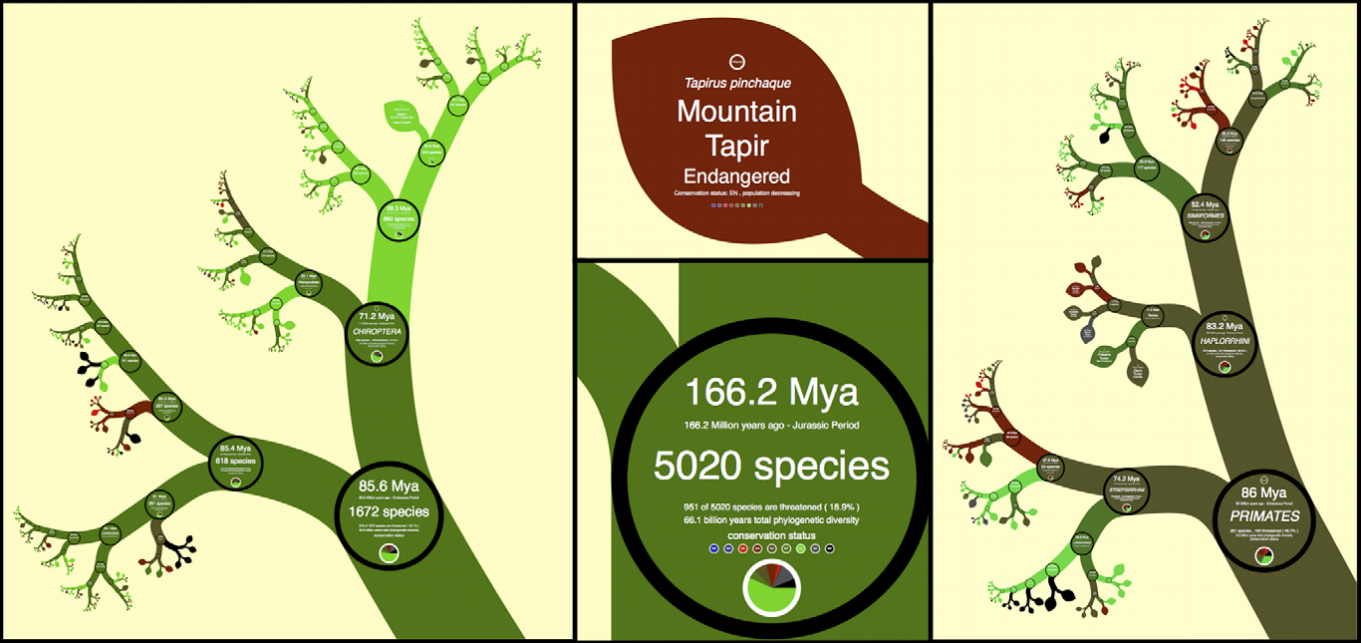
A mammal tree using data from [**8**] incorporating IUCN Red List metadata and common names [**13**].

There is no theoretical limit to the amount of metadata that can be put on a leaf or branch of the tree because these data can be displayed at any size and zoomed in on. Allowing branches to have a thickness assists the concept of scale when zooming and allows data such as graphs, maps, paragraphs of text, and images to be embedded inside branches and leaves. There are many further possibilities for using the colors and shapes of branches and leaves to reflect different metadata (Figure 4).

Zooming to reveal further details as implemented in OneZoom feels intuitive because it is akin to the way we explore the real world by moving closer to objects of interest to see them in greater detail. Data is always displayed in both intuitive and raw formats. For example, in the “natural” view of the tree of life (see Figure 3), the balance at each node (indicative of the ratio of species richness in each descendent branch) can be taken in at a glance from the thickness and angles of the branches, but the raw numbers can also be found by zooming in on the nodes. In OneZoom, we can be wasteful of space because there is an infinite quantity of space available. For example, structures that do not form a strict hierarchy, such as food webs, could be expressed by repetition of the same elements in multiple places. OneZoom is effective without fast processors, large high-resolution screens, stereoscopic vision, or multi touch inputs; however, these technologies, where available, could be straightforwardly exploited to provide an enhanced user experience.

## Visions for the Future

A significant issue with all tree visualization is that the ordering of descendent branches from any node and the positions in space of any node can be changed whilst still describing exactly the same tree [3]. This means that there are many ways to view a tree, each of which might emphasize different properties. We included three forms in the first release of OneZoom (see Figure 3), but all these have the common property that evolutionarily distinct species and clades appear larger. The natural view emphasizes tree balance over rates of diversification. We envisage the development of further forms for OneZoom in the future. For example, nodes could be aligned based on their dates using a non-linear timescale, with branches becoming progressively thinner towards the present day. This would complement the other forms by placing emphasis on diversification rates, timescales, and the species richness of clades; the tree would also then appear more like a familiar cladogram.

The concept of deep zooming for data presentation is not novel (see for example http://www.prezi.com), but we believe the idea has been under-utilized. We suggest that this may be because suitable methods for automatically laying out information on an infinite space are lacking; we hope that the OneZoom concept of using fractals will help resolve the problem. The fractal forms used within OneZoom need not look like trees, and further alternatives may be better and allow OneZoom to have applications outside of biology. For example, we speculate that an IFIG of the global financial markets could give an intuitive overview of the relative performance of each sector and subsector whilst allowing the more minute details to be revealed by zooming. Scientists exploring a scatter plot or color map could zoom in on any point or pixel to reveal further graphs, text, and information associated with just that data point. The files on a computer, the Internet, news, mind maps, genealogies, online stores, complex software structures, and industrial plants are all further examples of large and complex data sets that we imagine could be explored with OneZoom, even on a smartphone.

We most look forward to seeing OneZoom bring to life the remarkable and powerful phylogenetic datasets that many evolutionary biologists have strived to collect over recent years. We hope, together with a range of collaborators, to use OneZoom in the near future as a way to tackle the challenge of public education about evolution. For example, we envisage putting “microdots” on the branches of the tree, that when zoomed into, show fossil images and other evidence backing up the hypothesized evolutionary path of that branch. A richly annotated IFIG may help make the evidence, logic, and beauty of evolution easy to explore and understand in a way that is compelling and fun. The phylogenetic tree is also the most logical structure within which to explore the breadth of biodiversity on Earth, and so OneZoom could potentially be used to browse existing ecological databases of species such as the Encyclopedia of Life (http://eol.org/). These databases do face difficulties besides visualization, for example lack of available data and inconsistent naming of species. Nevertheless, we are confident that these problems will eventually be resolved simply by making gradual improvements to the many databases over a long period of time.

Our dream for the more distant future is an easily accessible web page presenting all that we know about life on Earth in one place. The logical way to do this is to build around the tree of life visualized using OneZoom; we may yet see the Google Maps equivalent for all life on earth.

## Supporting Information

Software S1 A self-contained version of the OneZoom software as a single html file. This file contains an embedded mammal tree using data from [8] and incorporates IUCN red list metadata and common names [13]. For further information we refer readers to the website www.onezoom.org, launched on the day of publication. Phylogeneticists are encouraged to download the latest editable version of OneZoom from the website and use it to create personalized IFIGs of their own data that can then be redistributed as supplementary material, in talks, and on the web. We welcome feedback on the software emailed to mail@onezoom.org.
(HTML)

Software S2 A self-contained version of the OneZoom software as a single html file (as Software S1). This file contains an embedded 408,135-tip phylogeny of small-subunit RNAs using data from SILVA [9] and is zipped to allow easy download.
(ZIP)

Text S1 Technical details of the algorithms used in OneZoom.
(PDF)

## Abbreviations

IFIG: interactive fractal-inspired graph
