# Supplementary material for: OneZoom: A Fractal Explorer for the Tree of Life
Source: PLoS Biol. 2012 Oct 16;10(10):e1001406. doi: 10.1371/journal.pbio.1001406 (PMC3472976; doi:10.1371/journal.pbio.1001406)
Supplement: Software S1 — A self-contained version of the OneZoom software as a single html file. This file contains an embedded mammal tree using data from [8] and incorporates IUCN red list metadata and common names [13]. For further information we refer readers to the website www.onezoom.org, launched on the day of publication. Phylogeneticists are encouraged to download the latest editable version of OneZoom from the website and use it to create personalized IFIGs of their own data that can then be redistributed as supplementary material, in talks, and on the web. We welcome feedback on the software emailed to mail@onezoom.org. (HTML) [file pbio.1001406.s001.htm]

 OneZoom - Everything on one page, all you have to do is zoom


 OneZoom - Tree of Life - 
Search
Grow
Options
Data
Reset
Tutorial
More
About
License

Look and Feel 
Detail -
Detail +
Zoom Level
View Type
Colour Scheme
Show Polytomies
 
Beginning
Reverse
Pause
Play
End
Faster
Slower
 
 Search 


 Latin 

 Common 

 Conservation 
Mark Results
Unmark Results
Show Results
Fly to Results
 Data in newick format, ultrametric, polytomies as branch length zero - 

Load data
