# Supplementary material for: OneZoom: A Fractal Explorer for the Tree of Life
Source: PLoS Biol. 2012 Oct 16;10(10):e1001406. doi: 10.1371/journal.pbio.1001406 (PMC3472976; doi:10.1371/journal.pbio.1001406)
Supplement: Text S1 — Technical details of the algorithms used in OneZoom. (PDF) [file pbio.1001406.s003.pdf]

Appendix A: Algorithm details

The basics of fractal plotting

One common method for plotting fractals that mimic the shapes of trees and plants is to use 'L-Systems'. The L-system technique involves a string of commands such as move forward, turn right and turn left. These commands define a path that, when plotted with a line, produce the desired fractal. The string of commands is 'grown' from an initial base string by iteratively replacing certain individual commands with strings of more complex ones. The greater the number of iterations, the more intricate the resulting shape will be. Mathematically speaking, the fractal is a theoretical object that appears as the number of iterations approaches infinity.

For example, to draw a simple fractal tree the base set of commands might define a trunk that splits into two branches. The iterative process would then replace each branch command with the command to draw a trunk and two branches, only at a smaller size. After a single iteration the image has a trunk, two medium branches and four small branches. After two iterations, it also has a further 8 very small branches and so on (see Figure A1). It is clear that this method could be modified to create a tree structure that could then be labeled with information and display a phylogeny. The lines of the fractal are of variable thickness so that the trunk of the tree is not only longer but also broader than the twigs. The text size scales proportionally with the width of the branches so that it

fits inside the tree itself rather than forming part of the background, we found this to be a more natural and visually pleasing way to embellish the tree shape with data. By giving the ability to zoom into the structure, information can be expanded and read that was initially invisible because its size was smaller than a pixel on the screen. For any split of a branch into two smaller branches, the width and length of the two smaller branches are defined as a function of the width and length of the larger branch. This results in a 'path' of information getting smaller and smaller that a user can follow intuitively by zooming in.

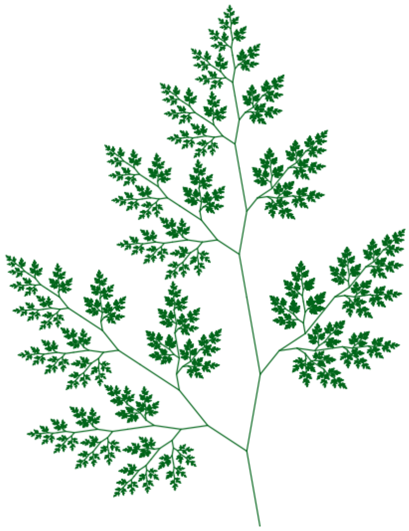

Figure A1: An example fractal that resembles plant growth – produced in R (R Development Core Team 2012) using only 17 lines of code.

The OneZoom plotting algorithms

Our algorithms do not explicitly store the string of commands that draws the fractal (as would be done in a classic L-system plot). Instead, we execute the drawing commands as they are created by using a recursive plotting function. This function repeatedly calls itself, using different parameters that create the more detailed parts of the image. To prevent the code from iterating indefinitely, we include an *if* statement that stops the drawing process when branches become too small to be visible at the current scale. We can illustrate the basic functionality of drawing trees in OneZoom with some pseudo code. The code has the following variables:

Parameters:

0 < ratioL < 1 , 0 < ratioR < 1 *(gives the length of the new left/right hand branches as a ratio with the current branch length)*

- π < angleL < π , - π < angleR < π *(gives the angle of the new left/right hand branch compared to the current branch. A zero indicates that it is just a continuation of the current branch)*

thickness *(gives the thickness to length ratio wanted for all lines)*

threshold > 0 *(gives the size of the smallest branch worth drawing, set at half a pixel for most detailed graphics, or set at larger values to increase plotting speed)*

Variables:

X , Y *(the coordinates of the path, initialised with the position of the base of the trunk, typically 0,0)*

angle *(the angle of the path's current direction where zero means vertically up, initialised with the angle of the tree, typically 0)*

length *(the length of the current branch, initialise with the size of tree required)*

Using these inputs, we can draw a tree.

```
Function: Draw_Tree ( X , Y , angle , length) {
        if (length > threshold ) {
                Draw branch of given length and angle, starting at X , Y
                        and having width given by (length * thickness)
                Update the values of X and Y to reflect the end of that
                        branch, rather than the start.
                Draw_Tree ( X , Y , angle+angleR , length * ratioR)
                Draw_Tree ( X , Y , angle+angleL , length * ratioL)
        }
}
```

The phylogenetic tree is stored as an object-oriented structure where each node contains a branch length, node name and two child node objects (which could be null if the node is a leaf). Given this storage method, the above algorithm becomes extremely efficient. A node has the ability to plot itself (which effectively plots the tree that descends from that node) by first drawing its descendent branch, and then asking its child nodes to plot themselves, passing down the appropriate parameters of position and scale. If a node is a leaf, then a

special plotting function draws a tip of the tree containing text in a leaf shape rather than drawing child nodes.

The angles between the branches, and the successive ratios of branch lengths can all be varied, and different possible choices give rise to distinct views of the tree. In OneZoom, there are three prewritten examples available, and a button allows users to quickly and easily switch between them. In all cases, we used the richness of the two clades at any split to decide which clade is drawn on the right hand branch and which on the left hand branch. The richer branch normally goes to the right (e.g. the tree is ladderised), but the direction (and the angles and ratios) can also work on an alternate basis to provide a different appearance.

One has to be extremely careful to select parameters (or functional forms) for the angles and ratios that do not cause the tree to be drawn with different parts overlapping one another. In OneZoom, non-overlap is guaranteed for all functional forms except those where the parameters are variable and set by the balance of the tree. Even in this case, non-overlap is very rare, and could be resolved in the future with more advanced algorithms. We expect future algorithms to be able to create other iteratively created tree structures with complementary properties to those of the three existing views.

Zooming, Translating and Optimisation

In OneZoom, we look at the IFIG through a ‘window’ so that anything outside the ‘window’ is not seen on screen and anything inside it is plotted. A larger ‘window’ would allow more of the IFIG to be displayed and thus graphics elements would appear smaller on screen as a result. Conversely, a smaller ‘window’ would mean only a tiny part of the IFIG is expanded to fill the display and graphics elements would appear larger on screen, but we would see fewer of them. OneZoom actually operates by keeping the ‘window’ stationary and of a fixed size so instead of moving and resizing the window, one equivalently moves or resizes the IFIG itself.

All the graphics elements that may need to be drawn in the IFIG can be associated to a particular node of the phylogenetic tree data structure. Each node typically encompasses some text, a section of branch expressed as a Bezier curve and a joint or tip expressed as a circle or leaf shape, this could be changed in the future to accommodate new and alternative plotting styles. Also stored in each node is a ‘horizon’, which corresponds to the smallest rectangle that could contain the graphics elements of that node and all its descendant nodes. The horizon is never drawn, but it speeds up the plotting process considerably as any node for which the horizon does not intersect the ‘window’ need not be considered further. The positions and parameters of all graphics elements including the horizon are pre-calculated in order to keep OneZoom as fast and responsive as possible after the initial load time.

The IFIGs corresponding to trees with thousands of tips are so large that conventional graphics techniques will not work - the precision of the numerical

calculations is not sufficiently accurate to pinpoint individual elements on such a large canvas. To resolve this problem, the positions and scales of all the graphics elements in any particular node of the tree are stored in reference to the parent node's position and scale, this does not require high precision as the parent node would not be far away or very different in size. The position and scale of the complete IFIG can thus be defined by the position and scale of any single chosen 'anchor' node against which all the others elements in the IFIG can be referenced. The node that we identify as the 'anchor' node changes dynamically during exploration of the IFIG so that it remains close to the 'window' and at a similar scale, otherwise numerical errors would again cause a problem.

The graphics elements in any part of an IFIG are only drawn if they are larger than a certain threshold size. Extra CPU power, if available, can be used to provide a more detailed view of the IFIG by decreasing the threshold, however the IFIG can be explored perfectly well with larger thresholds. Similarly, extra screen resolution allows more details of the IFIG to be displayed simultaneously but we have found the experience of exploring the IFIG to be acceptable on screen areas no larger than that found on a smart phone. The concept is thus very flexible for although it does not rely on emerging technologies to be effective, it can make use of them to enhance the user experience.

The image tiling method employed by Google maps could perhaps be used as an alternative to our algorithms here, but it seems more efficient to exploit the hierarchical structure of the graphics elements as a storage method and thus retain the benefits of an adjustable detail threshold. If we did use the tiling

method, our above algorithms would still have to be run to create the original tiles as vector graphics. There would be a large number of tiles and thus load times and memory requirements for processing newly inputted data would be significantly higher.

Smooth exploring with pre-fetching

The present IFIG examples load all the information into RAM when the page is opened. This takes just a few seconds for a 30,000-tip tree, but takes 3 minutes and 2Gb of RAM to load a 1.2 million tip dated tree with species names (On a 2009 model 13 inch MacBook Pro laptop). After the initial load time, exploration of the entire tree is completely smooth. Future versions of OneZoom could be improved so that only limited areas of the space were preloaded at a time and pre-calculations could be stored at the server level. This would be necessary to browse larger trees still where there are pictures and other metadata that need to be displayed in addition to the tree topology, it would also enable an almost instantaneous initial load time and use of the software on devices with less available RAM. However, even the current version of OneZoom can smoothly display very large phylogenetic trees. The main text includes screenshots from an IFIG of a 408,135-tip phylogeny.

Opening the IFIG

You can open the IFIG .html file in any web browser that supports the html5 canvas element. If you open the IFIG and see only buttons then your browser needs to be updated to use OneZoom. All modern mainstream browsers should

open the IFIG but the recommended browsers are: Safari (Mac), Firefox (Mac), Firefox (PC) and Google Chrome (PC). The tutorial button should (when pressed) give a full explanation of how to browse the IFIG in OneZoom

Data input specifications

To load your own data, the tree needs to be in Newick format, ultrametric, with branch lengths and with polytomies expressed as branches of length zero. Leaf names should be labeled with the convention 'Genus_species'. Naming of interior nodes is optional, but is fully supported. At the top of the html file (when opened in a text editor rather than a browser), there is a place to copy and paste your data together with comments that show how to customise the IFIG to your personal requirements so that it may become supplementary material for your own work. At present inputting your own trait data requires quite heavy editing of the code but we hope to make that easy in future versions of OneZoom.
